# Functional effects of 17alpha-hydroxyprogesterone caproate (17P) on human myometrial contractility in vitro

**DOI:** 10.1186/1477-7827-2-80

**Published:** 2004-12-07

**Authors:** Donal J Sexton, Michael W O'Reilly, Anne M Friel, John J Morrison

**Affiliations:** 1 Department of Obstetrics and Gynaecology, National University of Ireland Galway, Clinical Science Institute, University College Hospital, Newcastle Road, Galway, Ireland

## Abstract

**Background:**

17alpha-hydroxyprogesterone caproate (17P) administration reportedly improves outcome for women with a previous spontaneous preterm delivery. This study, using in vitro strips of human uterine smooth muscle, aimed to investigate the direct non-genomic effects of 17P on spontaneous and induced contractions in tissues obtained during pregnancy, and in the non-pregnant state.

**Methods:**

Biopsies of human myometrium were obtained at elective cesarean section, and from hysterectomy specimens, and dissected strips suspended for isometric recordings. The effects of 17P (1 nmol/L -10 micro mol/L) on spontaneous and agonist-induced (oxytocin 0.5 nmol/L for pregnant, phenylephrine 10 μmol/L for non-pregnant) contractions were measured. Integrals of contractile activity, including the mean maximal inhibition values (MMI) observed at the maximal concentration, were compared with those from simultaneously run control strips.

**Results:**

There was no significant direct effect exerted by 17P on pregnant or non-pregnant human myometrial contractility. The MMI ± SEM for spontaneous contractions in pregnant myometrium was 4.9% ± 7.2 (n = 6; P = 0.309) and for oxytocin-induced contractions was 2.2% ± 1.3 (n = 6; P = 0.128). For non-pregnant myometrium, the MMI ± SEM for spontaneous contractions was 8.8% ± 11.0 (n = 6; P = 0.121) and for phenylephrine induced contractions was -7.9% ± 6.5 (n = 6; P = 0.966).

**Conclusions:**

The putative benefits of 17P for preterm labor prevention are not achieved, even partially, by a direct utero-relaxant effect. These findings outline the possibility that genomic effects of 17P, achieved over long periods of administration, are required for its reported therapeutic benefits.

## Background

Preterm delivery constitutes a major problem in obstetric practice because of the large associated contribution to perinatal mortality and morbidity [1,2]. A significant proportion of all preterm deliveries are due to spontaneous preterm labor [2]. Despite much research effort, until recently, no effective method of preventing or treating preterm labor, and improving neonatal outcome, has been available. Meta-analysis of various tocolytic compounds in clinical practice has revealed that while they resulted in a delay in the interval to delivery, of time periods up to a week, they did not reduce the incidence of preterm delivery at different gestational ages [3]. More importantly, their use, in comparison to placebo, was not associated with any benefit in terms of objective measures of neonatal wellbeing or morbidity.

It has however been recently reported that weekly injections of 17-alpha-hydroxyprogesterone caproate (17P), in women who have had a previous spontaneous preterm delivery, significantly reduces the risk of preterm delivery before 37, 35 and 32 week's gestation [4]. In addition, infants of women treated with 17P had significantly lower rates of necrotising enterocolitis, intraventricular haemorrhage and the need for supplemental oxygen. While evidence for the use of progestational compounds for prevention of preterm delivery, and recurrent miscarriage, has been conflicting [5-7], meta-analysis restricted to trials of 17P has suggested a significant reduction in the preterm delivery rate. This reported benefit of 17P, while being a welcome development in therapeutic strategies for preterm labor, has raised many questions in relation to whether the same benefit would apply to low risk groups, and the potential effects, if any, from the castor-oil injection of placebo used [8,9]. One of the most important questions relates to the mechanism by which the drug works and there are currently no data to answer this. Progestins have the potential to exert both genomic and non-genomic effects. The aims of this study were focused specifically on the latter mechanism i.e. to investigate the direct effects of 17P on contractions of isolated human myometrium, both spontaneous and agonist-induced, in tissue obtained during pregnancy and in the non-pregnant state.

## Methods

### Patient Recruitment and Tissue collection

Patient recruitment took place in the Department of Obstetrics and Gynaecology, University College Hospital Galway. Ethical committee approval for tissue collection was obtained from the Research Ethics Committee at University College Hospital Galway and recruitment was by written informed consent. The biopsies were excised from the upper lip of the lower uterine segment incision in the midline i.e. upper portion of lower uterine segment. Women undergoing induction of labor were excluded from the study. For hysterectomy specimens, all women were pre-menopausal and undergoing abdominal hysterectomy without evidence of malignant uterine disease. Biopsies of myometrial tissue from hysterectomy specimens were obtained from the fundus. The biopsies were immediately placed in Krebs-Henseleit physiological salt solution (PSS), pH 7.4, containing: 4.7 mmol/L KCl, 118 mmol/L NaCl, 1.2 mmol/L Mg_2_SO_4_, 1.2 mmol/L CaCl_2_, 1.2 mmol/L KPO_4_, 25 mmol/L NaHCO_3_, and 11 mmol/L glucose. Tissues were stored at 4° C and used within 12 hours of collection.

### Tissue Bath Experiments

Longitudinal myometrial strips were dissected measuring approximately 2 × 2 × 10 mm. The strips were mounted for isometric recording under 2 grams of tension in organ tissue baths, as previously described [10-12]. The tissue baths contained 10 ml of Krebs-Henseleit physiologic salt solution maintained at 37°C, pH 7.4 and gassed continuously with 95%O_2_/5%CO_2_. Myometrial strips were allowed to equilibrate for at least 60 minutes, during which time the Krebs-Henseleit physiologic salt solution was changed every 15 minutes. After the equilibration period, regular spontaneous myometrial contractions were allowed to develop. In separate experiments contractions were induced by the addition of oxytocin (0.5 nmol/L) to pregnant myometrial strips, and phenylephrine (10 μmol/L) to strips obtained from non-pregnant myometrium.

Once regular phasic contractions had developed, the integrated tension for the first 20 minutes was calculated, and this value served as a control since no significant spontaneous reduction in myometrial contractility was observed over the duration of experiments in control strips (without addition of vehicle or 17P). The mechanical response of tissues was then measured by calculation of the integral of selected areas for 20 minute periods, corresponding to the cumulative exposure to 17P or vehicle, using the PowerLab hardware data acquisition system and Chart v3.6 software (AD Instruments, Hastings, UK). At 20 minute intervals 17P was added in a cumulative manner at concentrations of 1 nmol/L, 10 nmol/L, 100 nmol/L, 1 μmol/L, and 10 μmol/L (i.e., 1 × 10^-9 ^- 1 × 10^-5^M). Control strips (i.e. without exposure to 17P) were run simultaneously and for a similar duration, consisting of two separate groups of experiments as follows: 1. exposure of strips to PSS only; 2. exposure of strips to PSS and the vehicle for 17P. The overall duration of experiments was therefore 3 hours which is in accordance with standard in vitro myometrial experiments, allowing for an accurate drug exposure period of 20 minutes. The effects of 17P were assessed by subtracting the integrals of contractility measured after each bath exposure of 17P, from those obtained in control experiments (vehicle only). This allowed for calculation of the net effect of 17P on myometrial contractility. Potential effects of the vehicle were obtained by subtraction of the mean integrals measured after vehicle exposure, from those obtained without addition of vehicle i.e. spontaneous or agonist-induced contractions in PSS only. Percentage contractility was calculated by expressing the net integral measured after each 17P concentration addition, as a percentage of the integral calculated in the 20 minute period prior to any 17P addition. The mean maximal inhibition (MMI) refers to the mean percentage relaxation [i.e. 100% - mean contractility measured for each separate n sample of non-pregnant and pregnant myometrium] observed at the maximal bath concentration of 17P (i.e. 10 μmol/L), or corresponding concentration of vehicle for control strips.

### Drugs and Solutions

Oxytocin, phenylephrine and 17P were purchased from Sigma-Aldrich, Dublin, Ireland. Because 17P is a lipid soluble compound it was necessary to dissolve it in a lipophilic vehicle. The vehicle used consisted of 75% Dimethyl sulfoxide (DMSO): 25% ethanol, to make a 1 mmol/L (10^-3^M) stock solution of 17P. The resultant final tissue bath concentrations of DMSO and ethanol, at the maximum concentration of 17P investigated (i.e. 10 μmol/L or 10^-5^M), were 0.83% and 0.28% respectively, allowing for the cumulative effect at each bath addition. DMSO and ethanol were purchased from Sigma-Aldrich, Dublin, Ireland. Fresh Krebs-Henseleit physiological salt solution was made daily. Fresh 17P solutions were prepared on the day of experimentation and were maintained at room temperature for the duration of the experiment.

### Statistical Analysis

The integrals of contractile activity measured were compared to control values, and expressed as a percentage of that measured before drug addition. The effects of 17P on myometrial contractility were calculated for each 20-minute period of exposure from 1 nmol/L-10 μmol/L, and compared with the measurements obtained from control strips (ie. spontaneous, oxytocin-induced or phenylephrine-induced contractility, in the presence and absence of vehicle). The integrals of contractile activity were compared using a one-way ANOVA, which was followed by a Tukey HSD post-hoc. A value of P < 0.05 was accepted as statistical significance.

## Results

There were 6 women recruited for the study at the time of elective cesarean section. The reasons for cesarean section included previous cesarean section (n = 2), breech presentation (n = 2), unstable lie (n = 1) and postmaturity with poor cervical Bishop score (n = 1). The mean age, ± standard error of the mean (SEM), was 36.3 ± 2.8 years (range 26–46). The median gestation at the time of cesarean section was 39 weeks (range 38–41). In relation to parity, 2 of the women were para 0, 1 was para 1, and 3 were of parity greater than 1, at the time of recruitment. There were 6 women recruited at the time of abdominal hysterectomy. The reasons for hysterectomy included menorrhagia (n = 4), irregular vaginal bleeding (n = 1), and fibroids with an ovarian cyst (n = 1). The mean age ± SEM was 42.2 ± 2.4 years (range 36–50).

**Figure 1:**
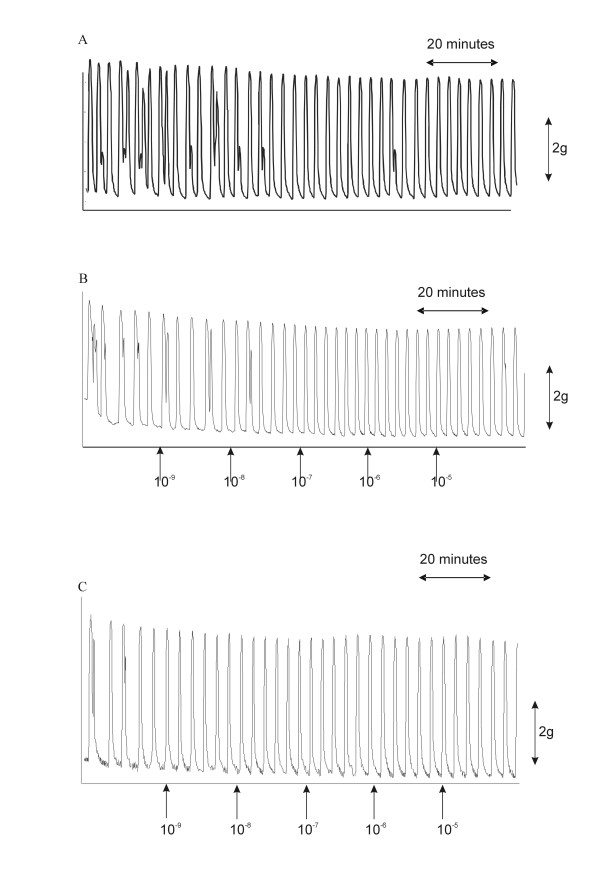
Effects of 17P on myometrial contractility in non-pregnant tissue. Representative recordings of spontaneous contractions in PSS only (A), the effects of cumulative additions of 17P vehicle (B), and the effects of cumulative additions of 17P (C) are shown.

For non-pregnant myometrial tissue representative recordings are shown in Figure 1. In Figure 1A a recording of spontaneous myometrial contractions is shown. In Figure 1B, the effects of vehicle for 17P on spontaneous contractions is demonstrated, and in Figure 1C the effects of addition of 17P are shown. The results of the calculated integrals are provided in Table 1. Bath exposure of the strips from hysterectomy specimens to 17P did not result in any alteration in contractile activity, at any of the bath concentrations studied, in comparison to vehicle only experiments (spontaneous contractions: P = 0.121; phenylephrine-induced contractions: P = 0.966).

**Table 1:** Mean Maximal Inhibition Values for Vehicle and 17P.

Myometrial Contractility	Non-Pregnant		Pregnant	
	Net Vehicle Relaxation ± SEM	Net 17P Relaxation ± SEM	Net Vehicle Relaxation ± SEM	Net 17P Relaxation ± SEM
Spontaneous	-0.6% ± 12.1 (n = 6)	8.8% ± 11.0 (n = 6)	49.7% ± 11.9 (n = 6)^♦^	4.9% ± 7.2 (n = 6)
*Agonist-Induced	31.8% ± 5.0 (n = 6)	-7.9% ± 6.5 (n = 6)	56.2% ± 2.3 (n = 6)^¶^	2.2% ± 1.3 (n = 6)

*Phenylephrine-induced in non-pregnant myometrium and oxytocin-induced in pregnant myometrium.

^♦ ^P = 0.022 in comparison to controls with PSS only

^¶ ^P < 0.001 in comparison to controls with PSS only

**Figure 2:**
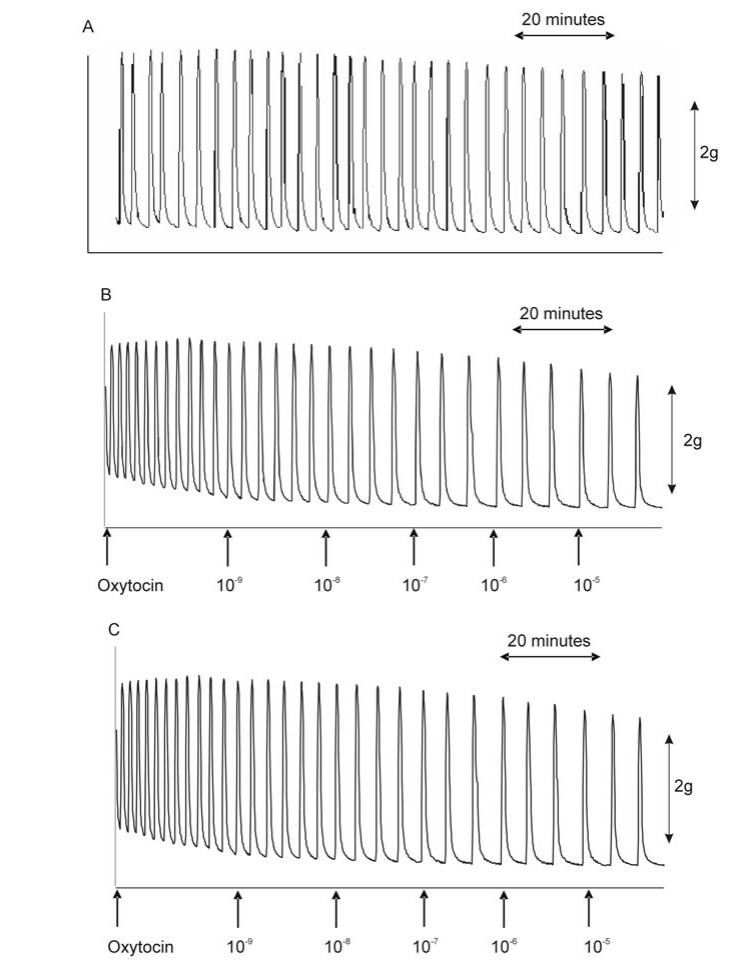
Effects of 17P on myometrial contractility in pregnant tissue. Representative recordings of oxytocin-induced contractions in PSS only (A), the effects of cumulative additions of 17P vehicle (B), and the effects of cumulative additions of 17P (C) are shown.

The results obtained from myometrium during pregnancy are similarly shown in Figure 2, and in Table 1. Figures 2A, 2B and 2C demonstrate recordings of oxytocin-induced contractions, the effects of vehicle, and the effects of 17P respectively. There was no significant net relaxant or uterotonic effect exerted by 17P on pregnant myometrial contractility, at any of the bath concentrations studied experiments (spontaneous contractions P = 0.309; oxytocin-induced contractions: P = 0.128). No significant difference was observed between the effects of 17P on contractility in either pregnant or non-pregnant myometrium.

The vehicle for 17P independently exerted a uterorelaxant effect on spontaneous and agonist-induced contractions in pregnant myometrial tissue only (spontaneous contractions P = 0.022; oxytocin-induced contractions: P = 0.000), and this was not observed in non-pregnant myometrial tissue (spontaneous contractions P = 0.241; phenylephrine-induced contractions: P = 0.068) (Table 1).

## Discussion

These results demonstrate that 17P does not appear to exert a direct relaxant effect on human myometrial contractions in vitro, in tissue obtained during the third trimester of pregnancy, or in the non-pregnant state. These findings are in contrast to the inhibitory effect of progesterone derivatives on spontaneous contractions in animal uterine tissues [13,14], and therefore suggest that the reported benefit of 17P in preventing preterm delivery in women who have had a previous preterm delivery, involves other mechanisms of action, presumably via its genomic effects. It seems likely that prolonged exposure of uterine smooth muscle to 17P, with resultant activation of the progesterone receptor isoforms, has the potential to modify gene transcription in order to maintain physiological uterine quiescence. However no direct effect on contractile activity was observed in our study over a period of hours of exposure.

Previous studies, using various progestins, have yielded conflicting results in terms of the direct effects of these metabolites on human myometrial contractility in vitro. Progesterone metabolites, in some studies, have been reported to decrease both the frequency and amplitude of contractions [15-17], while other reports have outlined that progesterone stimulates the frequency and tonus of contractions in term human myometrium [18,19]. It has been hypothesized that progesterone addition to myometrial strips only enhances contractility if the tissue specimen was never deprived of progesterone i.e. placed immediately in a medium containing progesterone [19]. The studies to date have included various progesterone metabolites but we are unaware of any previous studies evaluating the effects of 17P on human myometrial contractions in vitro. The focus on 17P in this study has arisen from the recently reported randomized clinical trial outlining its benefits clinically for recurrent preterm labor.

17P is a naturally occurring progesterone which has been isolated from corpus luteum and adrenal gland. The synthetic caproate ester, like the naturally occurring compound, is a steroid, is highly lipophilic and is inactive when administered orally. To achieve solubility for clinical studies, the vehicle used for injection was castor-oil [4]. This led to significant controversy in relation to the potential effect of castor-oil on myometrial contractions in the placebo arm of the recently reported study [8]. For laboratory, or in vitro studies, achieving the required solubility of progestins can also be a difficult process. Efforts to achieve solubility in previous reports have included the use of Hepes buffer and ultrasound baths [19,20] with a resultant maximum 50% solubility. After numerous attempts at solubility for 17P in our studies, the most appropriate vehicle was a combination of DMSO and ethanol. While both of these compounds may exert an effect on uterine contractility, the maximal bath concentrations of both solvents was 0.83% and 0.28% respectively, which is acceptable for in vitro studies of this nature. In addition, control experiments, with PSS only, and PSS plus vehicle, were simultaneously run, to clearly evaluate any potential effect of vehicle. It is however apparent from our results, that addition of vehicle only did exert a significant relaxant effect on myometrium obtained during pregnancy, but no effect of vehicle was observed in myometrium obtained in the non-pregnant state. For both tissues, addition of 17P did not alter contractile activity. While the vehicle effects observed were sizeable, the experiment design, the numbers of patients recruited, and the reproducibility of the results, clearly indicate that 17P does not exert a direct effect on human myometrial contractility. In addition, while the experiments described here investigated the effects of 17P added cumulatively, separate experiments (data not shown) using a single dose (the highest dose) revealed similar results i.e. there was no evidence of tachyphylaxis over the duration of the experiments.

There are some limitations to the methodology used in our study. The tissue biopsies from the non-pregnant uterus were obtained from the body of the uterus, while those obtained at the time of caesarean section were excised from the lower uterine segment. The results were similar for both tissue types, and there is reasonable evidence to suggest that the functional characteristics of lower and upper uterine segment myometrium are similar [21]. At present there are no data pertaining to differential progesterone receptor expression levels between upper and lower uterine segments. There are also obvious ethical constraints in obtaining biopsies from the upper segment of the uterus at cesarean section, and it is not feasible to dissect strips with certainty from the lower segment of the non-pregnant uterus. Secondly, the tissue samples obtained during pregnancy were all recruited from women at term. It could be that preterm myometrium displays a different responsiveness. Finally, in vivo administration of 17P could potentially result in the formation of a metabolite with a more efficient uterorelaxant effect.

## Conclusions

Whether 17P injections become incorporated for routine use in the management of preterm labor remains to be seen. Questions surrounding its true benefits, the associated difficulties in terms of vehicle solubility and placebo, and the mechanism of action, remain. Our findings highlight the solubility problems for scientific evaluation as occurred in clinical studies. Whatever the possible mechanism of action of 17P in reducing the incidence and adverse sequelae of preterm labor, this study demonstrates that it does not exert a direct relaxant effect, unlike other conventional methods of tocolysis investigated, and raised the likeliehood of a genomic effect secondary to long term administration during pregnancy.

## Authors' contributions

DJS performed the experiments and wrote the manuscript. MWO'R performed the experiments. AMF analysed the data and wrote the manuscript. JJM designed, supervised the study and wrote the manuscript. All authors read and approved the final manuscript.
